# Can electric fields explain inter-individual variability in transcranial direct current stimulation of the motor cortex?

**DOI:** 10.1038/s41598-018-37226-x

**Published:** 2019-01-24

**Authors:** Ilkka Laakso, Marko Mikkonen, Soichiro Koyama, Akimasa Hirata, Satoshi Tanaka

**Affiliations:** 1 Department of Electrical Engineering and Automation, Aalto University, Espoo, 02150 Finland; 2 Faculty of Rehabilitation, School of Health Sciences, Fujita Health University, Toyoake, 470-1192 Japan; 3 Department of Computer Science and Engineering, Nagoya Institute of Technology, Nagoya, 466-8555 Japan; 4 Laboratory of Psychology, Hamamatsu University School of Medicine, Hamamatsu, 431-3192 Japan

## Abstract

The effects of transcranial direct current stimulation (tDCS) on motor cortical excitability are highly variable between individuals. Inter-individual differences in the electric fields generated in the brain by tDCS might play a role in the variability. Here, we explored whether these fields are related to excitability changes following anodal tDCS of the primary motor cortex (M1). Motor evoked potentials (MEPs) were measured in 28 healthy subjects before and after 20 min sham or 1 mA anodal tDCS of right M1 in a double-blind crossover design. The electric fields were individually modelled based on magnetic resonance images. Statistical analysis indicated that the variability in the MEPs could be partly explained by the electric fields, subjects with the weakest and strongest fields tending to produce opposite changes in excitability. To explain the findings, we hypothesized that the likely locus of action was in the hand area of M1, and the effective electric field component was that in the direction normal to the cortical surface. Our results demonstrate that a large part of inter-individual variability in tDCS may be due to differences in the electric fields. If this is the case, electric field dosimetry could be useful for controlling the neuroplastic effects of tDCS.

## Introduction

Transcranial direct current stimulation (tDCS) is a widely used non-invasive method capable of eliciting changes in cortical excitability^1–3^. These neuroplastic changes have potential as a treatment for various psychiatric and neurological diseases that involve pathological changes in plasticity^4,5^. Cortical excitability changes induced by tDCS can be most reliably measured in the primary motor cortex (M1) using transcranial magnetic stimulation (TMS) to measure the amplitude of the motor evoked potentials (MEP)^6^. In such studies, the responses to tDCS have been found to be highly variable between individuals^7–11^. The underlying reasons of the variability are still unknown.

The physical agent of tDCS is thought to be the electric field (EF) that is generated in the brain and other tissues when direct current (usually 1–2 mA) is applied through electrodes attached to the scalp. The EF in the brain is weak, typically less than 1 V/m in strength^12–16^. Animal *in vitro* studies have shown that such weak EFs can affect the activity of M1^17,18^. Long-lasting excitability changes produced by weak EFs may depend on NMDA receptors^18^, which is also supported by electrophysiological studies in humans, where oral intake of NMDA antagonist suppressed the after-effects of tDCS^19^.

We have previously found that there are large differences in the EFs between individuals^15^. The differences are due to anatomical factors, such as gyral and sulcal anatomy as well as the volume of cerebrospinal fluid (CSF) and the thicknesses of the scalp and skull^14,15,20^. However, the role of EFs in inter-individual variability is still unclear. Are the effects of EF on neural tissue sufficiently similar in each individual so that EFs were useful for predicting the effects of tDCS? If they were, individual EF models could hypothetically be used to reduce variability and control the effects.

Here, we studied whether the EF was related to the after-effects of tDCS. We first performed an exploratory sham-controlled motor cortical tDCS study and individually calculated the EFs in all our subjects. In the experiments, we applied 1 mA anodal tDCS for 20 min on the right M1. Previously, a similar protocol has been shown to decrease the excitability of the right M1^21^. A similar inhibitory effect of long-duration anodal tDCS has also been shown using 26 min of 1 mA on the left M1^22^. Notably, halving the duration to 13 min enhanced the excitability^22^, consistently with the typical facilitatory effect of 9–13 min long anodal stimulation^23^.

To find which cortical sites are potentially affected by the EF, we decided to use partial least squares (PLS) regression^24,25^, which is an effective method for finding relationships between dependent variables (here: MEP amplitude) and a large number of collinear predictor variables (here: EF in the cortex). Compared to other commonly used approaches for feature extraction from imaging data, such as random field theory^26^, PLS regression was advantageous because we needed not define a region of interest *a priori*, which would have been arbitrary as we did not know in advance which site in M1 or other regions^27^ was affected by tDCS. At the potentially important cortical site, the data were further analysed using linear mixed effects models to investigate the direction and time-dependence of the effects.

## Methods

### Subjects

Twenty-eight subjects (7 females and 21 males; mean age ± SD = 27 ± 6 years; 26 right and 2 left handed) participated in the experiments. The subjects were the same who participated in our previous study^28^. The subjects were neurologically healthy and had no family history of epilepsy. The handedness was assessed using the Oldfield handedness questionnaire^29^. All subjects gave informed consent before participating in the experiments. The Human Ethics Committee at the National Institute for Physiological Sciences, Okazaki, Japan, approved the experiments. All methods were carried out in accordance with approved institutional guidelines and regulations.

### MRI

All subjects participated in MRI scanning. T1- and T2-weighted structural MRI scans of subjects participating were acquired using a 3.0 T MRI scanner (Verio; Siemens, Ltd., Erlangen, Germany). T1-weighted MRI were acquired using a Magnetization Prepared Rapid Acquisition in Gradient Echo (MPRAGE) sequence (TR/TE/TI/FA/FOV/voxel size/number of slices = 1800 ms/1.98 ms/800 ms/9°/256 mm/1.0 mm × 1.0 mm × 1.0 mm/176). T2-weighted MRI were acquired with the following parameters: TR/TE/FOV/voxel size/slice number = 4500 ms/368 ms/256 mm/1.0 mm × 1.0 mm × 1.0 mm/224 slices.

### Experimental parameters

**Figure 1:**
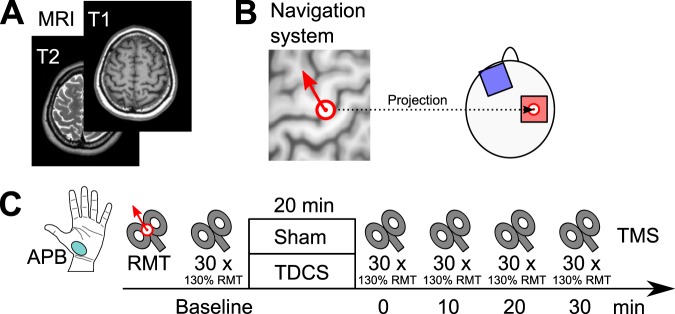
Experimental parameters. (**A**) T1- and T2-weighted MRI of each subject were acquired prior to the experiments. (**B**) In each subject, the location of the stimulation electrode was located above the centre of the hand knob, which was determined using a neuronavigation system. TMS was applied in the direction perpendicular to the central sulcus. (**C**) The effect of 20 min anodal tDCS or sham on the right motor cortex was monitored via TMS motor responses in the contralateral APB.

The experiment employed a double blind, sham-controlled, crossover design to study the effects of anodal tDCS over the right M1 on the MEPs. The experimental parameters are summarized in Fig. 1.

TDCS (1 mA) was applied using a DC STIMULATOR PLUS (neuroConn GmbH, Ilmenau, Germany) with two conditions. Conditions were separated by a washout period of at least three days. The MRI of each subject were acquired prior to the experiments.

The stimulation (anode) electrode (surface area 5 × 5 cm^2^) was placed over the hand M1 in the right hemisphere. The cathode (surface area 5 × 5 cm^2^) was placed over the contralateral orbit. Stimulation lasted for 20 min. In the sham condition, current (1 mA) was applied only for the first 15 s. The fade-in/fade-out time was 10 s in each condition. The subjects were asked to sit on a chair during the experiments, and the experimenter observed that the subject was maintaining rest.

The location of the anode was identified using an individual T1-weighted MRI and a frameless stereotaxic navigation system (Brainsight 2; Rogue Research Inc., Montreal, Canada). The experimenter first identified the “hand knob” structure of the precentral gyrus, the centre of which was projected to the scalp, which is illustrated in Fig. 1B. The centre of the anode was placed at the projected point on the scalp. TMS was also applied at the same point, in the direction perpendicular to the central sulcus, as identified using the navigation system.

As a measure of cortical excitability, MEPs were elicited using a Magstim 200^2^ magnetic stimulator (The Magstim Company Ltd, Whitland, UK). At the beginning of each condition, we determined the resting motor threshold (RMT). RMT was defined as the lowest stimulation intensity required for eliciting MEPs of 50 *μ*V peak-to-peak amplitude in five of ten trials in the fully relaxed left abductor pollicis brevis (APB) muscle^30^. The TMS coil was held in place manually, and its position was kept constant using the navigation system.

MEPs of the fully relaxed left APB muscle were recorded before and 0–30 min (with 10 min intervals) after tDCS. During test stimulation, TMS with the intensity of 130% of the RMT was applied 30 times for each time point. The TMS pulse interval was randomly assigned at 6, 7, 8, 9, or 10 s. Recorded electromyograms were amplified (1000x), band-pass filtered (10–1000 Hz), and sampled at 5 kHz. Finally, the mean peak-to-peak MEP amplitude was calculated.

### Anatomic models and inter-subject registration

T1- and T2-weighted MRI were segmented into distinct tissue compartments. Brain tissues were segmented using the FreeSurfer image analysis software^31–34^, and the remaining tissues were segmented using custom methods implemented in MATLAB (The MathWorks Inc., Natick, MA, US). The segmentation process and the tissue conductivities were identical to our previous studies^16,28^. The conductivities were (unit: S/m): grey matter 0.2, white matter 0.14, blood 0.7, compact bone 0.008, spongy bone 0.027, CSF 1.8, dura and muscle 0.16, skin and fat 0.08, and eye 1.5.

FreeSurfer with the default parameter values was used to generate a mapping from the surface of each individual subject’s brain to that of the standard brain; details of the procedure have been described earlier^16^. The standard brain was based on Montreal Neurological Institute (MNI) ICBM 2009a nonlinear asymmetric template^35,36^.

### Electric field modelling

The EFs calculated in each subject were identical to those reported in our previous study^28^. Briefly, the EFs were modelled using the following methods. The electrodes were modelled using a two-compartment model consisting of a 1 mm thick rubber pad (0.1 S/m) inserted in a 6 mm thick sponge saturated with physiological saline (1.6 S/m)^16^. The electrical sources were a current source (1 mA) and sink (−1 mA) placed inside the rubber pad of the anode and cathode, respectively.

The FEM with cubical 0.5 mm × 0.5 mm × 0.5 mm first-order elements was used to determine the electric scalar potential $\varphi$ from the Laplace-type equation $\nabla \cdot \sigma \nabla \varphi =0$. The equation was numerically solved using the geometric multigrid method^37^ to the relative residual of 10^−6^.

In each subject, the EF was calculated from $\overrightarrow{E}=-\,\nabla \varphi$ at the depth of 1 mm below the grey matter surface. We then calculated the absolute value (*E*_abs_) and the normal component of the EF (${E}_{{\rm{n}}}=\overrightarrow{n}\cdot \overrightarrow{E}$, where $\overrightarrow{n}$ is the inner normal vector of the cortical surface).

In order to compare the EFs from different subjects, *E*_abs_ and *E*_n_ were mapped to the MNI brain^16^. The final EFs were represented on a triangular surface mesh of the MNI brain, which consisted of 149319 vertices in the right hemisphere and 148076 vertices in the left hemisphere.

### Data analysis

MATLAB (version 2017b, The MathWorks, Inc.) was used for all statistical tests. The significance level was *P* < 0.05. Outliers were detected using Grubbs’ test.

#### Effects of Time, Session and EF on the MEP

We first analysed the experimental results without considering the EF, as would conventionally be done in tDCS studies. A linear mixed effects model was used to study the effects on the MEP normalized to the baseline. As fixed effects, we entered the effects of Time (*t* = 0, 10, 20, and 30 min after stimulation, denoted t0–t30), Session (real tDCS and sham) and their interaction effect. To account for the effects of the baseline MEP (MEP_base_) on the normalized MEPs, the fixed effects of MEP_base_, MEP_base_ × Time, MEP_base_ × Session, and MEP_base_ × Time × Session were also included in the model. By-subject intercepts were treated as random effects. Maximum likelihood estimation was used for determining the linear mixed effects model parameters.

To study the effect of the EF on the normalized MEPs, the absolute value and normal component of the EF were calculated at an observation point, ${\overrightarrow{r}}_{0}$ (to be defined later). We then added additional fixed effect terms for EF, Time × EF, Session × EF, and Time × Session × EF to the linear mixed effects model. The likelihood ratio test was used to test whether adding the EF terms improved the model significantly.

Using the linear mixed effects models, we studied the following questions: (1) was the mean value of the normalized MEP different from the baseline at any time point, (2) did the mean value depend on Session, (3) was the slope for MEP_base_ nonzero at any time point and (4) did it depend on Session, (5) was the slope for EF nonzero at any time point and (6) did it depend on Session, and (7) did any of the mean values or slopes depend on the time point? The coefTest function of MATLAB, which uses F-tests, was used for calculating the P-values. The Satterthwaite approximation was used for estimating the degrees of freedom. For visualization of the linear mixed effect models, we used a similar approach to calculate the P-values for each mean value, slope, and the difference in slopes between sham and real tDCS.

To test the effects of Session and EF on MEP_base_, we used paired two-tailed t-tests and/or Pearson correlation coefficients.

As a measure of the overall change in the cortical excitability, we calculated the mean MEP amplitude normalized to the baseline over post-stimulation time points (t0–t30)

1 $${\rm{mean}}\,{\rm{normalized}}\,{\rm{MEP}}=\mathop{{\rm{mean}}}\limits_{t}\,\frac{{\rm{MEP}}(t)}{{{\rm{MEP}}}_{{\rm{base}}}}.$$

Before this calculation, we verified that there were no significant differences between the post-stimulation time points.

We have previously found in the same subjects that the RMT correlated with the EF strengths in hand M1, and therefore had potential as a simple estimate of the EF strength^28^. To investigate whether the RMT was useful for estimating the normalized MEPs, we repeated the analysis replacing the EF with the RMT.

#### Estimation of important brain regions using PLS regression

We used PLS regression in MATLAB to study whether the measured MEPs could be explained using the calculated EFs, and, if yes, which brain regions were important for the prediction.

The input data to the PLS regression model were the following. The predictor variables, matrix **X** (28 × *n*), were the highest EF values (*E*_abs_ or *E*_n_) on the right hemisphere. The *n* vertices with the highest EF values were selected by calculating the (100 − *r*_E_)th percentile of the mean value of *E*_abs_ or |*E*_n_| over all 149319 vertices. To test the robustness of the approach, *r*_E_ was varied from 1 to 10%. The dependent variable, vector **Y** (28 × 1), was the mean normalized MEP. The columns of **X** were scaled by dividing them by their sample standard deviations and centred by subtracting their sample mean.

In the initial analysis, the number of PLS components (not to be confused with EF components) was varied from one to five, and the goodness of fit was measured in terms of *R*^2^ (multiple correlation coefficient) and *Q*^2^ (cross-validated *R*^2^). *R*^2^ and *Q*^2^ are the upper and lower bounds, respectively, of how well the model explains the data and predicts new observations^25^. To calculate *Q*^2^, we used 10-fold cross validation with 1000 Monte–Carlo repetitions. PLS component *i* was defined to be predictively significant if ${Q}_{i}^{2} > 1-{0.95}^{2}=0.0975$^38^. The analysis was also repeated for the sham MEP data. In this case, the EF should be unrelated to the MEP, and thus, no PLS components should have predictive significance.

After finding the number of significant components, we calculated the variable importance for the projection (VIP) to identify which brain regions were important for predicting MEPs from the EFs. Based on the important variables of PLS regression, we selected a single observation point, ${\overrightarrow{r}}_{0}$, in an anatomically relevant location to interpret the effect of the EF on the MEP amplitude using linear mixed effects models.

## Results

None of the participants reported side effects.

### Overall effect of tDCS on the MEPs

The effects of tDCS on the MEPs were initially analysed without the EFs. Analysis of MEP_base_ showed that the baselines of real tDCS (mean ± SD: 0.66 ± 0.26 mV, range: 0.21–1.15 mV) and sham (mean ± SD: 0.65 ± 0.32 mV, range: 0.27–1.39 mV) were not significantly different [paired t-test, *t*(26) = −0.197, *P* = 0.8]. Grubb’s test and visual inspection revealed one subject with exceptionally high MEP_base_ (real: 1.74 mV and sham: 1.53 mV). The subject was excluded from this and all further analyses.

**Figure 2:**
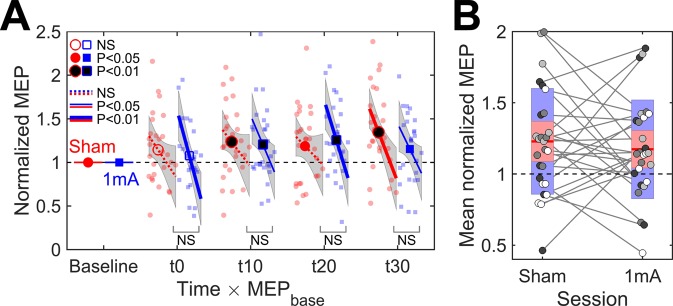
(**A**) Time course of change in the normalized MEP amplitude and its dependency on MEP_base_ (N = 27). Markers are the mean values, lines are the slopes for MEP_base_, grey regions are the 95% confidence intervals, and small markers are individual observations. Filled markers and solid lines indicate significant differences from the baseline. Bottom labels indicate the significance of the difference between the slopes of sham and real tDCS at each time point (NS = not significant). (**B**) Grand mean normalized MEP over post-stimulation time points. Disks represent the data for individual subjects. Shades of grey indicate the the division of the subjects to quartiles based on MEP_base_, lighter shades corresponding to higher values. Mean value is indicated by the horizontal line, and coloured bars represent the standard deviation (light blue) and 95% confidence interval (light red).

A linear mixed effect model with fixed effects of Time (t0–t30), Session (sham and real), MEP_base_, and their interactions was fitted to the normalized MEP data. The model is visualized in Fig. 2A. Grubb’s test for the model residuals indicated no outliers.

F-tests showed that the mean value of the normalized MEP differed significantly from the baseline [$F(8,98.2)=3.29$, $P=0.002$]. The difference was not significantly different between real tDCS and sham [$F(4,185.9)=1.58$, $P=0.2$]. Inspection of individual time points (Fig. 2A) showed that both sham and real tDCS increased the MEP amplitude compared to the baseline. MEP_base_ had a significant effect on the normalized MEPs [$F(8,175.7)=3.24$, $P=0.002$], subjects with a smaller baseline tending to show a larger increase in the MEP (Fig. 2A). The effect of MEP_base_ was not significantly different between real tDCS and sham [$F(4,189.8)=2.07$, $P=0.09$]. None of the mean values or slopes were significantly different between the time points [$F(12,185.9)=1.38$, $P=0.2$].

As the post-stimulation time points did not differ significantly, we calculated the mean MEP amplitude normalized to the baseline over all four post-stimulation time points to study individual differences in the responses to tDCS. The group-level as well as individual data are presented in Fig. 2B. The mean normalized MEPs did not have significantly different group-mean values [paired t-test, $t(26)=0.62$, $P=0.5$] nor significant correlation (Spearman $\rho =0.16$, $P=0.4$) between sham and real tDCS. Finally, we tested whether stimulation increased the difference between the MEPs of real tDCS and sham compared to the baseline, regardless of the direction of the difference. Wilcoxon signed-rank test showed that the absolute value of the difference between the MEPs of real tDCS and sham was significantly larger ($Z=-\,2.43$, $P=0.02$) after stimulation (median of mean over post-stimulation time points: 0.27 mV) than at the baseline (median: 0.18 mV).

Despite no significant differences between sham and real tDCS at the group level, these results indicated that stimulation had some effect, as the normalized MEPs of sham and real tDCS did not correlate within individuals, and the difference between the MEPs of sham and real tDCS after stimulation was larger than that expected from the baseline measurements. This could mean that the size and/or direction of the response differed between individuals. Were these differences due to chance or due to some systematic factor, such as the EF?

### Calculated EFs and PLS regression

**Figure 3:**
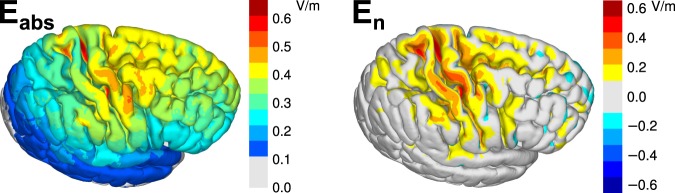
The absolute value and inner normal component of the EF averaged over 27 subjects. The EFs were first determined in each subject, registered to a common template, and finally averaged.

The absolute value and normal component of the EF were calculated individually in 27 subjects and registered to the standard brain. Figure 3 shows the average EFs in the right hemisphere.

PLS regression analysis with either *E*_abs_ or *E*_n_ as the predictor gave up to one predictively significant PLS component, depending on the percentage of the highest EF values used for the analysis (Table 1). Increasing the number of PLS components past one or using sham MEPs as dependent variables did not result in any predictively significant PLS components (Table 1, components 4 and 5 are not shown for brevity).

**Table 1:** Explained (${R}_{i}^{2}$) and predicted (${Q}_{i}^{2}$) variance of the first three PLS components (*i*).

Predictor	*r*_E_	${{\boldsymbol{R}}}_{{\bf{1}}}^{{\bf{2}}}$	${{\boldsymbol{R}}}_{{\bf{2}}}^{{\bf{2}}}$	${{\boldsymbol{R}}}_{{\bf{3}}}^{{\bf{2}}}$	${{\boldsymbol{Q}}}_{{\bf{1}}}^{{\bf{2}}}$	${{\boldsymbol{Q}}}_{{\bf{2}}}^{{\bf{2}}}$	${{\boldsymbol{Q}}}_{{\bf{3}}}^{{\bf{2}}}$
*Real tDCS*							
*E*_abs_	1%	0.39	0.44	0.12	0.17*	−0.53	−4.24
*E*_abs_	2%	0.39	0.48	0.10	0.12*	−0.79	−7.36
*E*_abs_	3%	0.39	0.50	0.08	0.09	−0.85	−9.38
*E*_abs_	10%	0.43	0.51	0.05	0.10*	−0.64	−15.93
*E*_n_	1%	0.54	0.32	0.11	0.12*	−0.99	−5.19
*E*_n_	2%	0.62	0.32	0.05	0.12*	−1.40	−12.19
*E*_n_	3%	0.67	0.28	0.04	0.09	−1.94	−19.39
*E*_n_	10%	0.87	0.12	0.01	−0.00	−7.04	−88.15
*Sham*							
*E*_abs_	1%	0.23	0.50	0.17	−0.23	−1.33	−5.61
*E*_abs_	2%	0.30	0.51	0.15	−0.28	−1.46	−8.27
*E*_abs_	3%	0.33	0.51	0.14	−0.32	−1.58	−9.85
*E*_abs_	10%	0.39	0.54	0.06	−0.38	−1.67	−20.76
*E*_n_	1%	0.58	0.25	0.12	−0.32	−2.60	−8.17
*E*_n_	2%	0.66	0.23	0.09	−0.25	−3.21	−12.70
*E*_n_	3%	0.71	0.22	0.06	−0.23	−3.88	−18.91
*E*_n_	10%	0.89	0.10	0.01	−0.13	−9.03	−122.36

The predictor variables are the EF absolute value or the normal component and the dependent variables are the mean normalized MEP of either real tDCS or sham stimulation. Ratio *r*_E_ indicates the percentage of the highest EF values used for the PLS regression. *Q^2^ > 0.0975.

**Figure 4:**
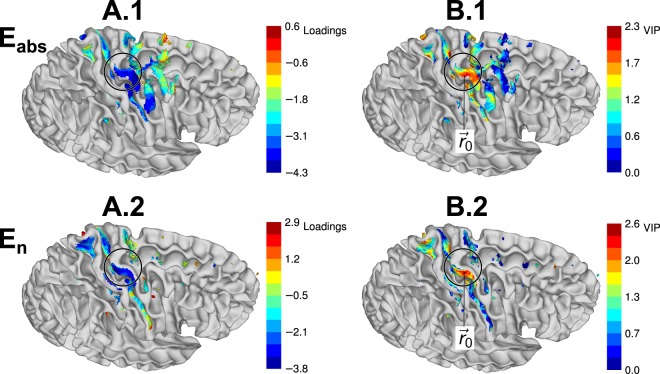
Important brain regions estimated using PLS regression analysis. (**A**.**1** and **2**) Loadings of the first PLS component for the absolute value (**A**.**1**) and normal component (**A**.**2**) of the EF. (**B**.**1** and **2**) VIP, indicating the relative importance of brain regions for predicting the mean normalized MEP from the absolute value (**B**.**1**) or the normal component (**B**.**2**). ${\overrightarrow{r}}_{0}$ indicates the point with the maximum VIP for the normal component. Coloured regions are the cortical regions that contain the highest 2% EFs and that were used as predictors for PLS regression. The surface of the white matter is shown in grey. Circle indicates the inverted omega of the hand knob.

The models with one PLS component were used for the subsequent analysis. The loadings of the PLS regression model with *r*_E_ = 2% are visualized in Fig. 4A. PLS score plots indicated no violations of homogeneity or curvature of the data. Normal probability plots were used to verify the normality of residuals, and no outliers were detected in residual plots either by visual inspection or by Grubbs’ test.

Next, we investigated which brain regions were important for predicting the mean normalized MEPs using the VIP as the measure. The regions with high VIP in Fig. 4B are candidates for the site of action where the EF has an effect on the MEPs. For the subsequent analysis, we selected the point with the maximum VIP as an observation point, ${\overrightarrow{r}}_{0}$, as shown in Fig. 4B. The same point was the global maximum for *r*_E_ ≤ 5% and a local maximum for larger *r*_E_. In MNI coordinates, ${\overrightarrow{r}}_{0}=(42,-\,13,66)$. Based on the probabilistic cytoarchitectonic atlas of FreeSurfer, ${\overrightarrow{r}}_{0}$ is located at the border between Brodmann areas 6 and 4a. The choice of ${\overrightarrow{r}}_{0}$ from among several candidates is motivated by the fact that ${\overrightarrow{r}}_{0}$ is close to the TMS hotspot of the ABP muscle projected to the cortex, (−41 ± 4, −16 ± 4, 60 ± 4), measured in the left hemisphere^39^.

### Effects of the EF

Next, we investigated how the EFs at ${\overrightarrow{r}}_{0}$ affect the normalized MEPs and their time course. The summary statistics of ${E}_{{\rm{abs}}}({\overrightarrow{r}}_{0})$ were mean ± SD: 0.46 ± 0.14 V/m and range: 0.24–0.79 V/m. For ${E}_{{\rm{n}}}({\overrightarrow{r}}_{0})$, the summary statistics were mean ± SD: 0.39 ± 0.12 V/m and range: 0.20–0.60 V/m. The EFs correlated strongly (Pearson $r=0.89$), indicating that the EF was approximately normal to the cortical surface at ${\overrightarrow{r}}_{0}$. For this reason, we chose to focus on ${E}_{{\rm{n}}}({\overrightarrow{r}}_{0})$ in the following analysis.

First, we tested the baseline effects of EF. The Pearson correlation coefficients between ${E}_{{\rm{n}}}({\overrightarrow{r}}_{0})$ and MEP_base_ were $r=0.15$ ($P=0.4$) and $r=0.26$ ($P=0.2$) for sham and real tDCS, respectively. The regression slopes were not significantly different between sham and real tDCS [$t(50)=-\,0.20$, $P=0.8$].

**Figure 5:**
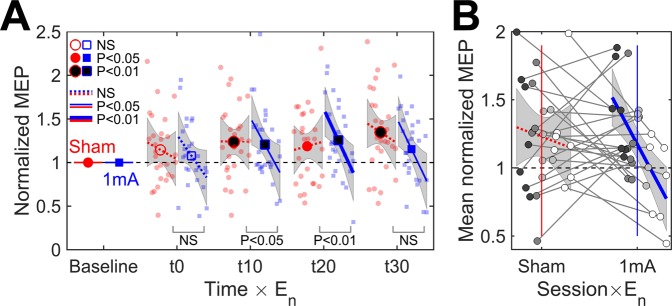
(**A**) Effect of the EF normal component and time on normalized MEP for 20 min 1 mA anodal tDCS of the right M1 (N = 27). Lines and shaded areas are the regression lines (range: 0.20–0.60 V/m) and the 95% confidence intervals. Filled markers and solid lines indicate significant differences from the baseline in the mean values and slopes, respectively. Bottom labels indicate the significance of the difference between the slopes of sham and real tDCS at each time point (NS = not significant). (**B**) Simple linear regression between ${E}_{{\rm{n}}}({\overrightarrow{r}}_{0})$ and grand mean normalized MEP. Disks show the data for individual subjects, different shades of grey indicate the division of subjects into quartiles based on their EFs. The grey line segments indicate the changes between sessions. Coloured vertical lines show the mean values of ${E}_{{\rm{n}}}({\overrightarrow{r}}_{0})$.

${E}_{{\rm{n}}}({\overrightarrow{r}}_{0})$ and its interactions with Time and Session were input into a linear mixed effects model as fixed effects. Adding the terms with ${E}_{{\rm{n}}}({\overrightarrow{r}}_{0})$ into the model improved the model significantly compared to the model without the effects of the EF (likelihood ratio test, ${\chi }^{2}(8)=17.87$, $P=0.02$). Figure 5A visualizes the fitted linear mixed effects model. Grubb’s test for the model residuals detected one outlier data point (exceptionally low normalized MEP at t0 in the subject with the lowest EF). Inclusion or exclusion of the outlier did not change the conclusions, and therefore we have included it in the analysis.

F-tests showed that, similarly to the model without the EF, the mean values of normalized MEPs changed significantly from the baseline [$F(8,96.4)=3.78$, $P=0.0007$], and the change depended on MEP_base_ [$F(8,173.0)=2.46$, $P=0.02$]. Neither effect differed significantly between sessions [$F(4,185.1)=1.71$, $P=0.1$, and $F(4,189.6)=1.36$, $P=0.2$, respectively]. ${E}_{{\rm{n}}}({\overrightarrow{r}}_{0})$ had a significant effect on the normalized MEP [$F(8,98.0)=2.35$, $P=0.02$], and the effect of ${E}_{{\rm{n}}}({\overrightarrow{r}}_{0})$ was significantly different for real tDCS and sham [$F(4,185.6)=3.63$, $P=0.007$]. Investigation of the slopes for ${E}_{{\rm{n}}}({\overrightarrow{r}}_{0})$ showed that real tDCS tended to have more negative slopes than that at baseline or those of sham (Fig. 5A). The slopes for sham were not significantly different from the baseline at any time point.

Time did not have significant effects on any of the intercepts or slopes [$F(18,185.0)=1.11$, $P=0.3$]. Therefore, we used simple linear regression to characterize the effect of ${E}_{{\rm{n}}}({\overrightarrow{r}}_{0})$ on the mean normalized MEP. The fitted linear model (Pearson $r=-\,0.63$, $P=0.0005$) for real tDCS was

2 $${\rm{mean}}\,{\rm{normalized}}\,{\rm{MEP}}\approx 1.17-0.72\frac{{E}_{n}({\overrightarrow{r}}_{0})-m}{m},$$

where $m=0.39$ V/m is the sample mean of ${E}_{{\rm{n}}}({\overrightarrow{r}}_{0})$. The 95% confidence intervals of the intercept and slope were $[1.06,1.28]$ and $[\,-\,1.09,-\,0.35]$, respectively. The regression slope for sham stimulation was not significant ($r=-\,0.12$, $P=0.6$). The partial correlation coefficients without the confounding effect of MEP_base_ were $r=-\,0.59$ ($P=0.001$) and $r=-\,0.09$ ($P=0.6$) for real tDCS and sham, respectively. Figure 5B visualizes the linear regression model as well as the individual data. It can be seen that the individuals with the largest EFs showed decreased MEPs compared to sham, whereas the subjects with the lowest EFs showed either absence of effect or increased MEP compared to sham.

### Effects of the RMT

Similarly to our previous study in the same subjects^28^, both ${E}_{{\rm{n}}}({\overrightarrow{r}}_{0})$ (Pearson $r=-\,0.64$, $P=0.0003$) and ${E}_{{\rm{abs}}}({\overrightarrow{r}}_{0})$ ($r=-\,0.70$, $P=0.00004$) strongly correlated with the RMT (average of two measurements).

To study whether the RMT could be used instead of the EF to explain the changes in the normalized MEPs, we replaced ${E}_{{\rm{n}}}({\overrightarrow{r}}_{0})$ with the RMT in the linear mixed effects model. Analysis of the model coefficients showed no significant effects of the RMT at any time point [$F(8,99.2)=0.59$, $P=0.8$] nor significant differences in the effects of the RMT between real tDCS and sham [$F(4,184.6)=0.52$, $P=0.7$]. The RMT tended to correlate with MEP_base_ (real tDCS: $r=-\,0.35$, $P=0.07$, and sham: $r=-\,0.38$, $P=0.05$).

Simple linear regression showed that the RMT weakly correlated with the mean normalized MEPs of real tDCS ($r=0.43$, $P=0.03$; partial correlation without the confounding effect of MEP_base_: $r=0.35$, $P=0.07$). After removing the effect of ${E}_{{\rm{n}}}({\overrightarrow{r}}_{0})$, the partial correlation between the RMT and the mean normalized MEP disappeared ($r=0.04$, $P=0.8$), indicating that their correlation was due to the EF. For sham, the correlation coefficient between the RMT and the mean normalized MEP was $r=0.25$ ($P=0.2$), and the partial correlations without MEP_base_ and the EF were $r=0.20$ ($P=0.3$) and $r=0.23$ ($P=0.2$), respectively.

## Discussion

We studied the effect of 20 min 1 mA anodal tDCS on the excitability of the right M1, and modelled the EF in each individual subject. The initial findings suggested no significant differences between sham and real tDCS in the group-level MEPs. However, the responses to sham and real tDCS differed at the individual level. Using regression analysis, we showed that individual differences in the EF could partly explain the variability in responses.

If the EF has an effect on the individual MEPs, from which brain regions does the effect originate from? We explored this question using PLS regression to find relationships between the absolute value or normal component of the EF and the MEPs. The results showed that it was possible to predict a part of the effect using linear regression involving the EF normal component. Furthermore, the EF values in the hand area were important for the prediction. While these results are not a direct proof of the exact origin of the effects, the hypothesis that the possible effect originated from the hand M1 is attractive due to several reasons. Firstly, the sites with high importance were located in the lateral part of the hand knob at the anterior bank of the central sulcus [MNI coordinate (±42, −13,66)], which is at the border between the primary motor cortex (BA4a) and premotor areas (BA6). This is in the immediate vicinity of the TMS hotspot of the studied APB muscle^39^, which could explain why the EFs at this site are important for predicting the changes in MEPs. Furthermore, the direction of the EF was approximately normal to the cortical surface. In previous *in vitro* studies, EFs applied in the normal direction have been shown to produce long lasting after-effects^17,18^. Therefore, a plausible hypothesis for explaining our findings is that the after-effects of tDCS are mediated by the normal component of the EF at or near the TMS hotspot. However, this needs to be confirmed in additional studies.

Linear mixed effects models were used to analyse how *E*_n_ in hand M1 affected the MEPs. The results showed that tDCS, but not sham, changed the slope between the EF and MEP amplitude, indicating that subjects with low and high EFs responded differently to stimulation. TDCS changed the slope to a more negative direction, i.e., subjects with a stronger *E*_n_ exhibited a larger decrease (or smaller increase) in the MEP compared to sham or baseline than subjects with a weaker *E*_n_. A possible explanation for the negative effect of the EF is that, as shown in previous studies, long duration (≥20 min) 1 mA anodal tDCS may decrease the motor cortical excitability^21,22^. The negative slope is consistent with these findings; the inhibitory effect is stronger in individuals with a stronger EF and absent in subjects experiencing weak fields in M1. However, the effects of tDCS are known to be non-linearly dependent on the current intensity^40–42^ and stimulation duration^22^. Therefore, our findings should not be extrapolated to other experimental conditions.

As far as we know, our study is the first to combine individual EF modelling with tDCS experiments. However, a few recent studies have used EF models to design and analyse the results of tDCS experiments. The effects of different EF components were studied previously by Rawji *et al*.^43^, who investigated the excitability changes using two electrode montages that produced EFs dominantly in the posterior-anterior (PA) or medial-lateral (ML) directions (left M1, 1 mA, 10 min, FDI muscle, N = 15). They found that a larger EF in the PA direction decreased the excitability, whereas the EF in the ML direction did not^43^. Our experiments are not directly comparable due to different target muscle, duration, and electrode montage. However, common to both studies was that the effective EF component might have been the normal component, *E*_n_, which depends on the representation of the target muscle in the curved surface of the hand area. In another study, Fischer *et al*.^27^ showed that a multifocal tDCS montage that produced a weaker EF in the left M1 resulted in a greater increase in excitability than conventional tDCS (2 mA, 10 min, FDI muscle, N = 15). Consequently, they argued that the effects of tDCS on the motor cortical excitability likely originated from regions outside M1^27^. Although obtained using different stimulation parameters, our results and those of Rawji *et al*.^43^ show that the EF strength–response relationship can also be negative, and thus, the findings of Fischer *et al*.^27^ could also be explained by a local effect of EF in M1.

Existing tDCS protocols that have been used since the early 2000s typically apply the same input current to all subjects^6,23,44^. This approach may be problematic, as our results suggested that the subjects with the lowest and highest EF strengths may respond oppositely to the same input current. Therefore, the findings at the group level may become weak or not significant. Indeed, in our experiment, no significant group-level differences compared to sham or baseline would have been found without considering the inter-individual differences in the EF. For comparison, several previous studies have also reported small group-level responses but high inter-individual variability^7–9^. If our results generalize to other stimulation parameters, EF models could be used to select the stimulation current individually, which could reduce variability.

Separately from the effect of the EF, we found that MEP_base_ had a significant effect on the normalized MEPs. The effect was similar for both sham and real tDCS: subjects with a higher MEP_base_ tended to decrease the MEP, and subjects with a lower baseline tended to increase the MEP. Wiethoff *et al*.^7^ have also reported a similar negative effect. We believe that the effect directly follows from normalization and is unrelated to tDCS: If the MEP were a random variable, the conditional expectation of the normalized MEP would be a decreasing function of MEP_base_.

Despite the correlation between the RMT and the EF^28^, we found that the RMT had relatively weak effects on the after-effects of tDCS. The correlation coefficient between the RMT and mean normalized MEP was $r=0.43$, which was only marginally better than that obtained for sham. The correlation disappeared when the confounding effect of the EF was excluded, indicating that the RMT and and the post-stimulation MEPs were linked solely through the EF. Interestingly, a correlation of similar strength but with the opposite sign ($r=-\,0.47$) has been reported previously for 15 min 1 mA anodal tDCS^42^.

Due to the exploratory nature, our study has several limitations that should be considered in future studies. Firstly, we only considered a single stimulation current (directly proportional to the EF) in each subject. Using additional EFs in each subject could have been used to confirm the regression slopes and take into account inter-individual differences in the sensitivity to EF in linear mixed effects models. If multiple currents are used in future studies, non-linearities^40–42^ should also be considered when selecting the current values. Secondly, repeating the measurements for each condition would have allowed incorporating intra-individual variability in the statistical models. Notably, previous studies have shown relatively low intra-individual variability for anodal TDCS^11,45^. Thirdly, only one electrode configuration was used in each subject. More than one electrode configuration would have increased the amount of input data to the PLS regression model and would have possibly improved the prediction of the affected regions. Fourthly, we unexpectedly found a significant facilitatory effect of sham stimulation. We are unsure of the reasons, as other recent sham-controlled studies have not shown any significant effects of sham^11,42,43^. The effect of sham persisted at least 30 min after stimulation, which highlights the importance of sham control in future studies. Future studies can also select the observation point or region of interest *a priori*. The observation point found in this study is useful for the APB muscle of the left hand. For other targets, the TMS hotspot projected on the cortex may be a reasonable choice. Our results can also be used to estimate the required sample size for future studies. Based on simple linear regression between the EF and normalized MEP, a reasonable estimate for the coefficient of determination is approximately 0.35, which would require at least 20 data points (subjects) to ensure a statistical power of 80%.

In conclusion, this exploratory study showed that individually calculated EFs were related to inter-individual differences in the responses to tDCS. A potential hypothesis for explaining our findings is that the individual effects of tDCS are mediated by the normal component of the EF in the hand area of M1, at or close to the TMS hotspot. If the effect is confirmed, EF modelling could be the key for reducing inter-individual variability in tDCS.

## Data Availability

The datasets generated during the current study are available from the corresponding author on reasonable request.
